# Establishment of a Prognostic Signature of Stromal/Immune-Related Genes for Gastric Adenocarcinoma Based on ESTIMATE Algorithm

**DOI:** 10.3389/fcell.2021.752023

**Published:** 2021-11-24

**Authors:** Shan Yu, Yan Wang, Ke Peng, Minzhi Lyu, Fenglin Liu, Tianshu Liu

**Affiliations:** ^1^ Department of Medical Oncology, Zhongshan Hospital, Fudan University, Shanghai, China; ^2^ Department of Biostatistics, Zhongshan Hospital, Fudan University, Shanghai, China; ^3^ Center of Evidence-Based Medicine, Fudan University, Shanghai, China; ^4^ Department of General Surgery, Zhongshan Hospital, Fudan University, Shanghai, China

**Keywords:** gastric cancer, tumor microenvironment, immune checkpoint inhibitors, risk scores, prognosis 2

## Abstract

Different subtypes of gastric cancer differentially respond to immune checkpoint inhibitors (ICI). This study aimed to investigate whether the Estimation of STromal and Immune cells in Malignant Tumor tissues using Expression data (ESTIMATE) algorithm is related to the classification and prognosis of gastric cancer and to establish an ESTIMATE-based gene signature to predict the prognosis for patients. The immune/stromal scores of 388 gastric cancer patients from TCGA were used in this analysis. The upregulated differentially expressed genes (DEGs) in patients with high stromal/immune scores were identified. The immune-related hub DEGs were selected based on protein-protein interaction (PPI) analysis. The prognostic values of the hub DEGs were evaluated in the TCGA dataset and validated in the GSE15460 dataset using the Kaplan-Meier curves. A prognostic signature was built using the hub DEGs by Cox proportional hazards model, and the accuracy was assessed using receiver operating characteristic (ROC) analysis. Different subtypes of gastric cancer had significantly different immune/stromal scores. High stromal scores but not immune scores were significantly associated with short overall survivals of TCGA patients. Nine hub DEGs were identified in PPI analysisThe expression of these hub DEG negatively correlated with the overall survival in the TCGA cohort, which was validated in the GSE15460 cohort. A 9-gene prognostic signature was constructed. The risk factor of patients was calculated by this signature. High-risk patients had significantly shorter overall survival than low-risk patients. ROC analysis showed that the prognostic model accurately identified high-risk individuals within different time frames. We established an effective 9-gene-based risk signature to predict the prognosis of gastric cancer patients, providing guidance for prognostic stratification.

## Introduction

Gastric cancer, with gastric adenocarcinoma comprising 95% of the total cases, ranks fifth for incidence and fourth for mortality worldwide (Sung et al., 2021). Currently, curative gastrectomy with or without perioperative chemotherapy remains a standard treatment for gastric cancer. For unresectable or metastatic gastric cancer, chemotherapy using drugs such as platinum and taxol is a standard treatment, with trastuzumab, a monoclonal antibody against human epidermal growth factor receptor 2 (HER2), as a first-line treatment for HER2-positive patients and ramucirumab, a monoclonal antibody against vascular endothelial growth factor receptor 2, as a second-line treatment for advanced disease (Bang et al., 2010; Sasako et al., 2011; Wagner et al., 2017). Recently, immune checkpoint inhibitors (ICI) against programmed cell death 1, programmed cell death-ligand 1, and cytotoxic T-lymphocyte antigen 4 have emerged as a novel therapeutic strategy for various types of advanced cancer, including gastric cancer (Kang et al., 2017). However, ICI monotherapy remains the third-line treatment for advanced, heavily pretreated gastric cancer due to its moderate efficacy in patients (Muro et al., 2016; Fuchs et al., 2018). It is necessary to investigate the mechanism underlying the limited efficacy of ICI in gastric cancer treatment.

In 2014, the Cancer Genome Atlas (TCGA) has classified gastric adenocarcinoma into four subtypes, including Epstein-Barr virus (EBV)-positive tumors, microsatellite instable (MSI) tumors, tumors with chromosomal instability (CIN), and genomically stable (GS) tumors (2014). By comparing the tumor microenvironment (TME) in the four subtypes, Derks et al. have found that compared with other subtypes, the GS subtype has enriched CD4^+^ T cells, macrophages, and B cells, along with tertiary lymphoid structures (TLS) in approximately 50% of cases (Derks et al., 2020). TLS within solid tumors is considered an antitumor school for inflammatory T-cells, B cells, and dendritic cells as well as an antibody factory to combat cancer (Colbeck et al., 2017). Different components of TLS, such as mature dendritic cells, chemokines, T cells, or B cells, are correlated with favorable clinical outcomes of patients with solid tumors (Dieu-Nosjean et al., 2014). Therefore, GS gastric cancer patients are promising candidates for immunotherapy. However, bench-top and clinical studies have shown that compared with EBV and MSI tumors, GS tumors have poorer responses to ICI (Derks et al., 2016; Kim et al., 2018). We hypothesized that the distinct immune environment of different subtypes of gastric cancer might explain their differential responses to ICI.

If this hypothesis is true, stromal scores or immune scores are related to ICI therapy, MSI or EBV subtypes which has been prove to be sensitive to ICI therapy should have lowest stromal scores. But accrding to this figure, stromal scores in MSI or EBV subgroup are moderate.

The TME refers to the cellular environment where tumors exist, comprising various cellular components, including endothelial cells, immune cells, and stromal cells (Hanahan and Coussens, 2012; Arneth, 2019). Infiltrating stromal and immune cells are major nontumor constituents of TME, being closely associated with the prognosis of cancer patients (Sato et al., 2005; Mlecnik et al., 2011; Miyoshi et al., 2016; Blom et al., 2019). To predict the fraction of immune and stromal components in tumor samples, Yoshihara et al. have developed an ESTIMATE algorithm using gene signatures of immune and stromal cells (Yoshihara et al., 2013). Investigators have successfully applied the ESTIMATE algorithm to identify immune/stromal-related genes that predict prognosis in different cancers, such as glioblastoma, colon cancer, and pancreatic adenocarcinoma (Alonso et al., 2017; Jia et al., 2018; Meng et al., 2020). However, the immune/stromal-related genes with prognostic value in gastric adenocarcinoma remain largely unknown.

In this study, to identify stromal/immune-related genes that are possibly related to the response of gastric cancer patients to ICI therapy, we sorted 388 patients with gastric adenocarcinoma from The Cancer Genome Atlas Research Network, 2014 database into high and low stromal/immune score groups according to the ESTIMATE algorithm and performed bioinformatical analyses. We identified 9 stromal/immune-related differentially expressed genes (DEGs) that predicted poor prognosis of gastric cancer patients, providing new information and future research direction for ICI therapy in gastric cancer.

## Materials and Methods

### Sample Collection

The gene expression profiles and clinical characteristics of 388 patients (Table 1) with gastric adenocarcinoma were obtained from the TCGA database (https://tcga-data.nci.nih.gov/tcga/) as a training cohort. The immune and stromal scores of each sample were acquired from the Estimation of STromal and Immune cells in MAlignant Tumor tissues using Expression data (ESTIMATE; http://bioinformatics.mdanderson.org/estimate/) (Yoshihara et al., 2013). The GSE15460 dataset was downloaded from the Gene Expression Omnibus (GEO; https://www.ncbi.nlm.nih.gov/geo/) as a validation cohort.

**TABLE 1:** Clinical characteristics and immune/stromal scores of patients with gastric adenocarcinoma in the TCGA database.

Variables	*n* (%)	Immune scores (‾X ± S)	Stromal scores (‾X ± S)
Gender			
Male	252 (65)	—	—
Female	136 (35)	—	—
TCGA classification			
EBV	30 (7.7)	2,672 ± 246.4	831.3 ± 243.4
MSI	73 (18.8)	1,488 ± 144.2	447.4 ± 155.9
CIN	223 (57.5)	847.7 ± 89.95	460.1 ± 95.5
GS	50 (18.9)	2,133 ± 188.8	1977 ± 179.4
Lauren classification			
Diffuse type	66 (17.0)	2,283 ± 148.4	2,117 ± 149.8
Intestinal type	181 (46.6)	1,141 ± 103.9	490.5 ± 94
ACRG classification			
MSI	79 (20.4)	1,442 ± 139.3	398.2 ± 152.3
MSS	276 (71.1)	1,387 ± 92.9	794.3 ± 92.1

TCGA: The Cancer Genome Atlas; EBV: Epstein-Barr virus; MSI: microsatellite instable; CIN: chromosomal instability; GS: genomically stable; MSS: microsatellite stable.

### Identification, Annotation, and Enrichment Analysis of DEGs

DEGs were identified using the limma package of R language. The genes with | Log2 fold-change (FC) |> 1.5 and *p-*value less than 0.001 were considered DEGs. Heat map and clustering were generated using ClustVis. Gene ontology (GO) and Kyoto Encyclopedia of Genes and Genomes (KEGG) analyses were performed using DAVID (https://david.ncifcrf.gov/). A *p* value <0.05 was considered statistically significant.

### Construction of Protein-Protein Interaction (PPI) Network

PPI was analyzed using the Search Tool for the Retrieval of interaction Genes database (STRING, https://string-db.org/). A PPI network was established using Cytoscape GeneMANIA, followed by the modular analysis of the first layer of the hub genes.

### Survival Analysis

Patients were categorized into high- and low-expression groups according to the mean mRNA levels of corresponding genes. Survival analysis was performed using Kaplan-Meier curves and log-rank test. Statistical analysis was conducted using the Survival of R language. A *p* value <0.05 was considered statistically significant.

### The Cox Proportional Hazards Model

A prognostic risk signature was established based on the Cox proportional hazards model using the R package of Survival. The risk score of each patient was calculated based on the Cox coefficient and expression level of each gene: (Kim et al., 2012). The TCGA patients were categorized into low-risk and high-risk groups according to the mean risk score. The Kaplan-Meier curves were generated. Log-rank analysis was used to assess the prognostic value of the risk score. A time-dependent receiver operating characteristic (ROC) curve analysis was performed, and the area under the curve (AUC) was calculated to assess the accuracy of the prognostic risk signature for the prediction of time-dependent cancer death.

### Statistical Analysis

Box plots and contingency plots were generated using Prism 5.0 (GraphPad, San Diego, CA, United States). Other than the mentioned above, statistical analysis was conducted using two tails paired Student’s *t*-test or Chi-square test. A *p* value <0.05 was considered statistically significant.

## Results

### Immune and Stromal Scores Are Associated With the Classification and Prognosis of Gastric Adenocarcinoma

The gene expression profiles of 388 patients with gastric adenocarcinoma were obtained from the TCGA database. The clinical characteristics of the patients were summarized in Table 1. The tumors were divided into different subtypes according to different classification criteria. The samples without corresponding information for classification were excluded. The stromal scores ranged between −3,120.7 and 4,033, and the immune scores ranged between −2,378.4 and 4,908.6.

**FIGURE 1:**
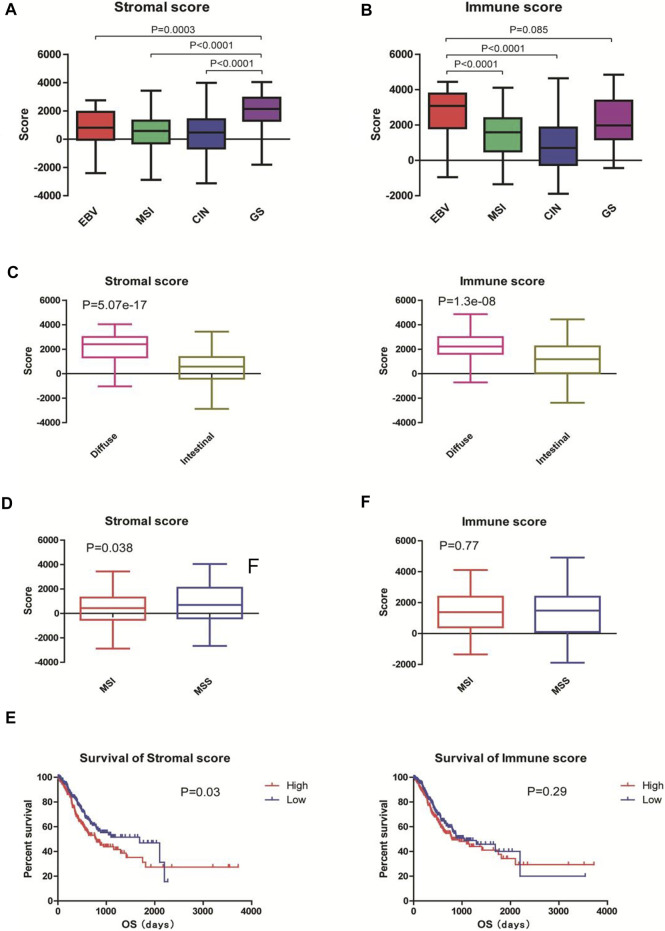
Immune and stromal scores were associated with the classification and prognosis of gastric adenocarcinoma. The gene expression profiles and clinical characteristics of 388 patients with gastric adenocarcinoma were obtained from the TCGA database. The immune and stromal scores of each sample were acquired from the Estimation of STromal and Immune cells in MAlignant Tumor tissues using Expression data (ESTIMATE; http://bioinformatics.mdanderson.org/estimate). **(A,B)** Tumors were classified into EBV (*n* = 30), MSI (*n* = 73), CIN (*n* = 223), and GS (*n* = 50) subtypes according to TCGA classification in 2014. The stromal scores and immune scores are shown. **(C)** Tumors were classified into intestinal-type (*n* = 66) and diffuse-type (*n* = 181) tumors according to the Lauren classification. The stromal scores and immune scores are shown. **(D)** Tumors were classified into MSI (*n* = 79) and MSS (*n* = 276) tumors according to the Asian Cancer Research Group classification. The stromal scores and immune scores are shown. EBV: Epstein-Barr virus; MSI: microsatellite instable; CIN: chromosomal instability; GS: genomically stable; MSS: microsatellite stable. **(E)** Patients were divided into high- (*n* = 196) and low-score (*n* = 192) groups according to the mean stromal and immune scores. Kaplan-Meier survival analysis was performed to examine the correlations of stromal scores (left) and immune scores (right) with the overall survival of patients.

According to the latest TCGA classification (2014), 30 samples (7.7%) were EBV-positive tumors, 73 (18.8%) were MSI tumors, 223 (57.5%) were tumors with CIN, and 50 (18.9%) were GS tumors. The stromal scores were significantly different among the subgroups in descending order of GS, EBV, MSI, and CIN (all *p* < 0.001 vs. GS; Figure 1A). The immune scores were different among the subgroups in descending order of EBV, GS, MSI (*p* < 0.001 vs. EBV), and CIN (*p* < 0.001 vs. EBV). No significant difference was observed in the immune scores between EBV and GS tumors (*p* > 0.05; Figure 1B).

According to the Lauren classification (Lauren, 1965), 66 (17.0%) samples were intestinal-type tumors and 181 (46.6%) were diffuse-type tumors. The diffuse-type tumors exhibited significantly higher stromal and immune scores than the intestinal-type tumors (both *p* < 0.001; Figure 1C).

According to the Asian Cancer Research Group (ACRG) classification (Cristescu et al., 2015), 79 (20.4%) patients were MSI tumors and 276 (71.1%) were microsatellite stable (MSS) tumors. The MSI subtype had significantly lower stromal scores than the MSS subtype (*p* < 0.05; Figure 1D, left). No significant difference was observed between the immune scores between MSI and MSS subgroups (*p* > 0.05; Figure 1D, right).

To examine the correlations of stromal and immune scores with the prognosis of gastric cancer patients, we divided the 388 patients into high- and low-score groups according to the mean stromal and immune scores and performed survival analysis. The Kaplan-Meier curves showed that patients with low stromal scores had longer overall survivals than those with high stromal scores (1,686days vs. 779 days; *p* = 0.03; Figure 1E, left). However, no statistical significance was observed between patients with low immune scores and those with high immune scores (1,403days vs. 794 days; *p* = 0.29; Figure 1E, right). Taken together, these results suggest that stromal and immune scores are associated with the classification of gastric adenocarcinoma and that high stromal seems to predict the poor prognosis of patients.

### Identification of DEGs in Patients With High Stromal and Immune Scores

**FIGURE 2:**
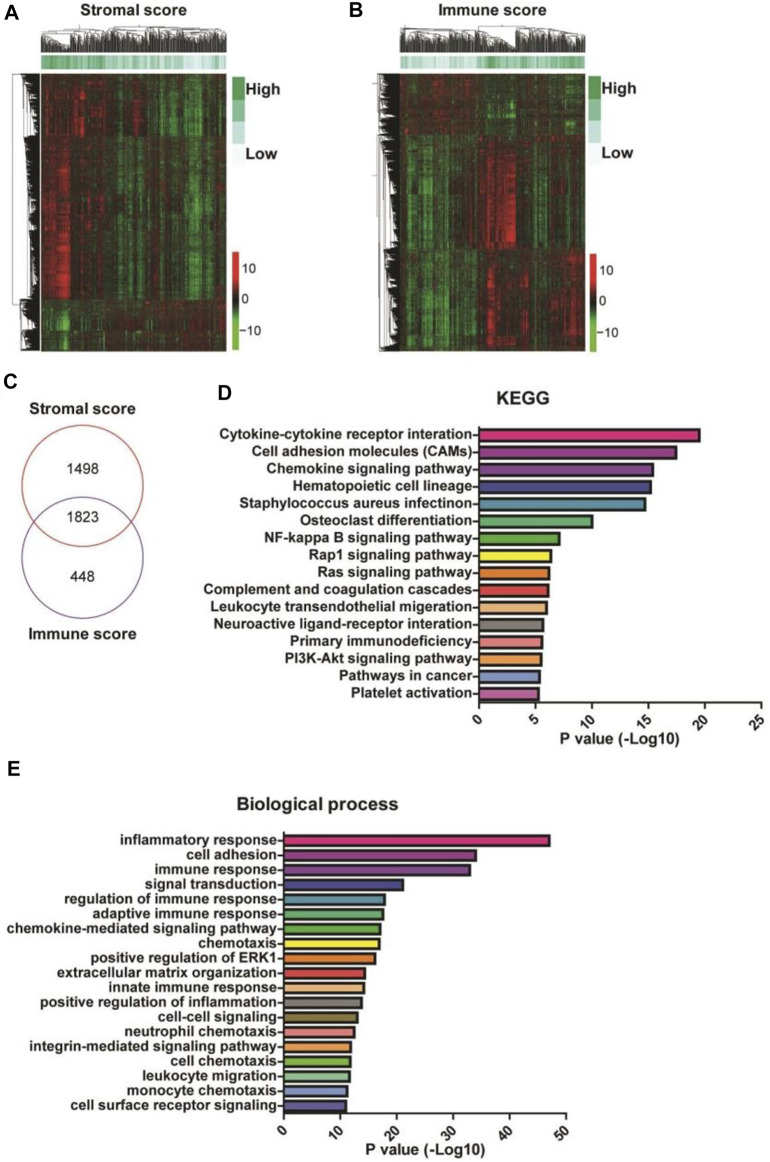
Identification and enrichment analysis of differentially expressed genes (DEGs) in gastric cancer patients with high stromal and immune scores. **(A,B)** The heatmaps of the DEGs (|Log2 fold-change| > 1.5 and adjusted *p*-value < 0.001) in gastric cancer patients with high stromal (left) and immune (right) scores. **(C)** A Venn diagram depicts 1823 upregulated DEGs in the intersection between high stromal and high immune scores-related DEGs. **(D,E)** Functional enrichment analysis of the DEGs was performed using Gene Ontology (GO) terms and Kyoto Encyclopedia of Genes and Genomes (KEGG) pathways.

To identify the genes related to high stromal and immune scores that might predict the poor prognosis of patients with gastric cancer, we compared the gene expression profiles of the 388 TCGA samples. We obtained 3,321 upregulated DEGs in patients with high stromal scores (Figure 2A, left) and 2,271 upregulated DEGs in patients with high immune scores (Figure 2A, right), compared with patients with low stromal or immune scores. A total of 1823 DEGs were at the intersection (Figure 2C). KEGG analysis revealed that the 1823 DEGs were mainly enriched in cytokine-cytokine receptor interaction, cell adhesion molecules, and chemokine signaling pathway (Figure 2D). GO annotation showed that the 1823 DEGs were mainly involved in inflammatory response, cell adhesion, and immune response (Figure 2E).

### The mRNA Levels of Hub DEGs Negatively Correlate With the Overall Survival of Patients With Gastric Adenocarcinoma

**FIGURE 3:**
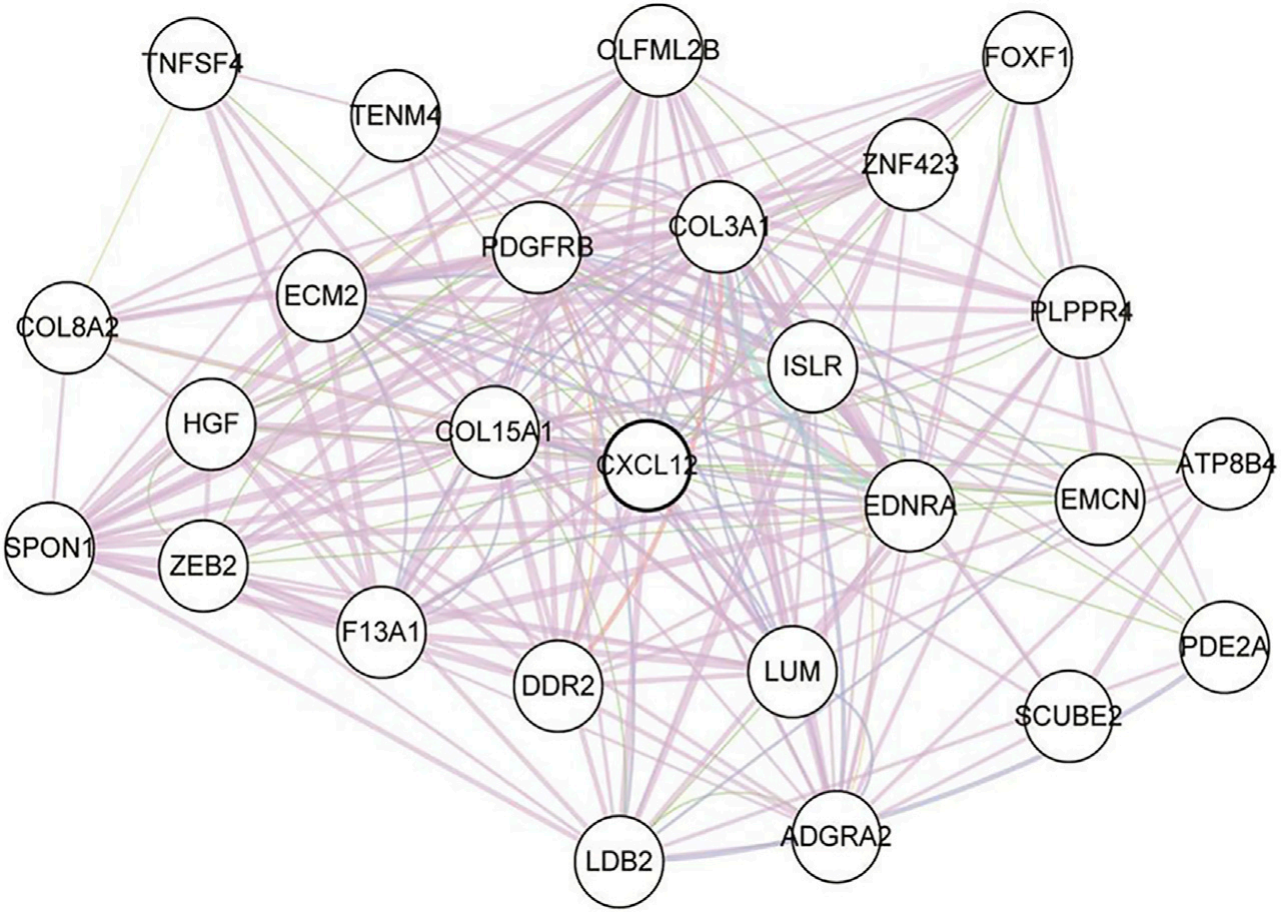
The protein-protein interaction graph of the CXCL12 module. Nodes represent proteins, and lines represent the possible relationships. CXCL12: C-X-C motif chemokine ligand 12.

**FIGURE 4:**
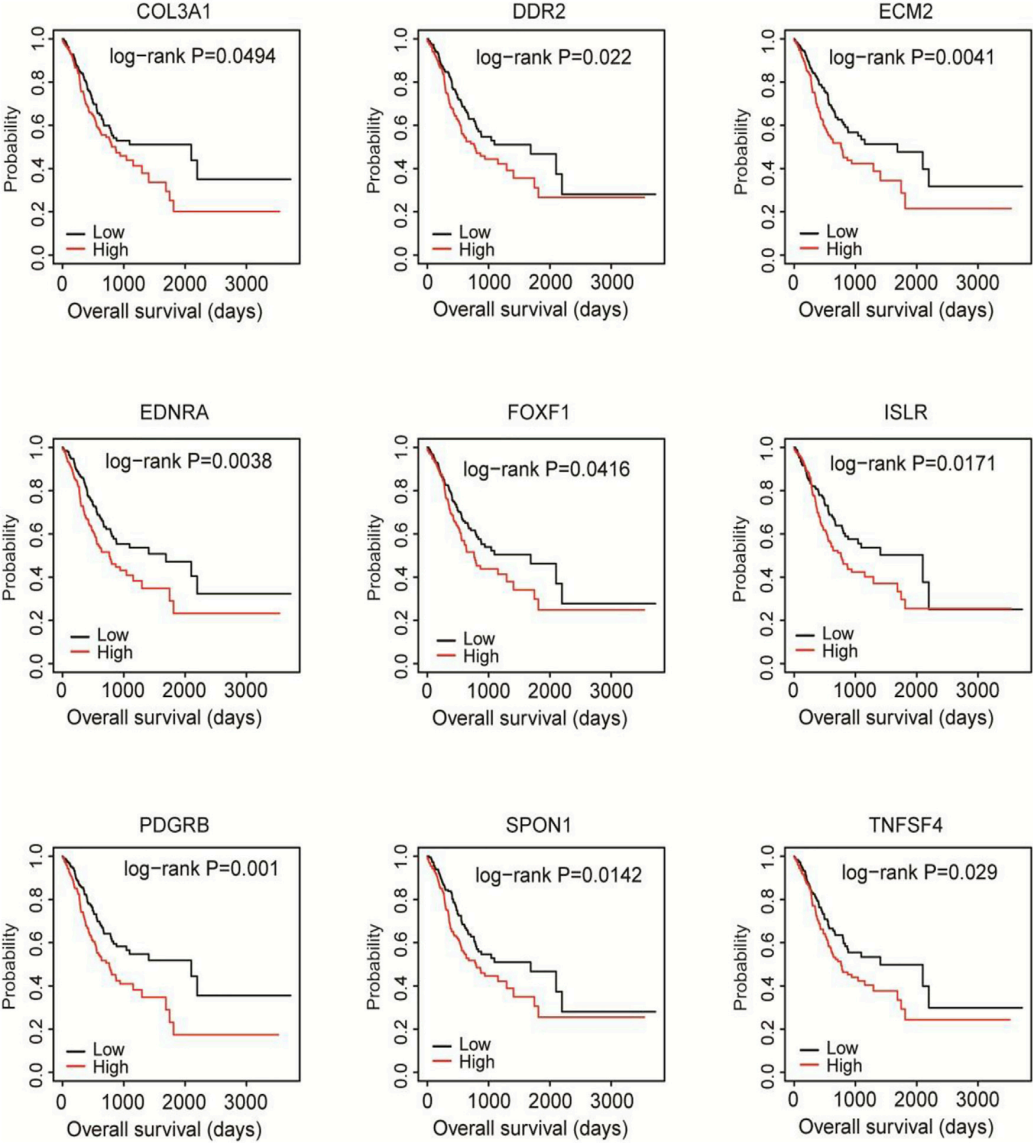
Kaplan-Meier survival analysis of the associations of 9 hub DEGs with the overall survival of the TCGA cohort. Patients were divided into high and low expression groups according to the mean mRNA level of each hub DEG. Kaplan-Meier survival analysis was performed.

To find out the hub DEGs in the 1823 candidates, we established a PPI network. Because we aimed to identify stromal/immune-related genes, we further analyzed the CXCL12 module that was mostly associated with immune response and inflammation in the PPI network. Why only choose CXCL12 here, other immune related moduls? Figure 3 demonstrates 26 hub genes (nodes) in the module, including immune-related ISLR (Nagasawa et al., 1997) and PDGFRB (Demoulin and Montano-Almendras, 2012), as well as stromal related-COL3A1 (Wang et al., 2016), COL15A1 (Egusa et al., 2007) LUM (Vij et al., 2004), and ECM2 (Malik et al., 2015). To evaluate the prognostic values of the hub genes in CXCL12 module, we performed survival analysis. The Kaplan-Meier curves showed that the mRNA levels of 9 genes, including COL3A1, DDR2, ECM2, EDNRA, FOXF1, ISLR, PDGFRB, SPON1, and TNFSF4, were significantly, negatively correlated with the overall survival of the TCGA patients (all *p* < 0.05, Figure 4).

**FIGURE 5:**
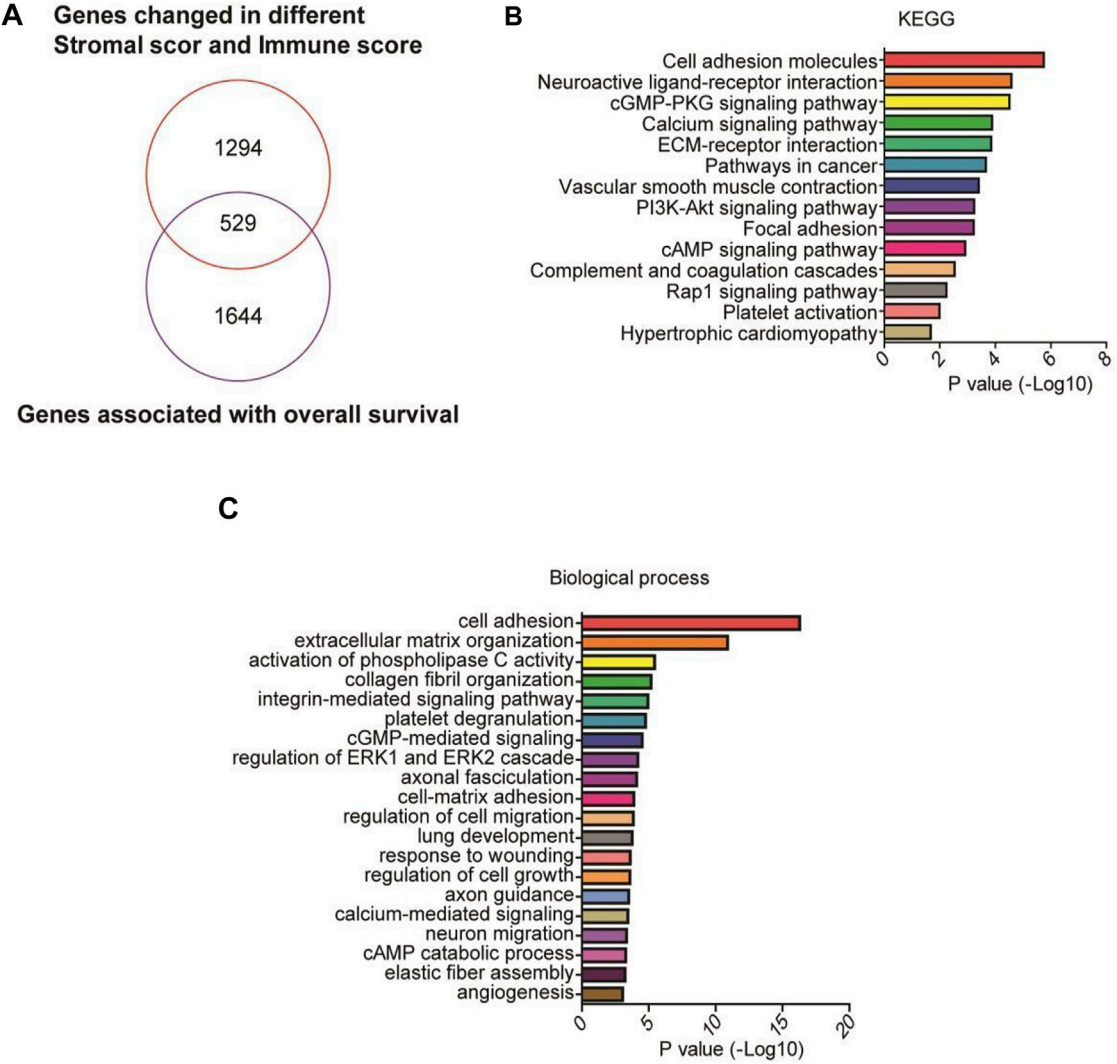
GO and KEGG analyses of prognostic genes. **(A)** A Venn diagram depicts 529 DEGs in the intersection between 2,173 prognosis-related genes and 1823 upregulated high stromal/immune scores-related DEGs in the TCGA cohort, including the 9 genes that were negatively correlated with the overall survival of the patients. **(B,C)** Functional enrichment analysis of the 529 DEGs was performed using GO terms and KEGG pathways.

We further identified 2,173 prognosis-related genes in the TCGA cohort. A total of 529 genes were at the intersection between the 1823 DEGs and the 2,173 prognosis-related genes (Figure 5A), including the 9 genes that were negatively correlated with the overall survival of the patients. KEGG analysis revealed that the 529 DEGs were mainly enriched in cell adhesion molecules and neuroactive ligand-receptor interaction (Figure 5B). GO annotation analysis showed that the 529 genes were mainly involved in cell adhesion, extracellular matrix organization, and collagen fibril organization (Figure 5C).

### Validation of the Prognostic Value of the 9 Hub Genes and Establishment of a Prognostic Risk Signature

**FIGURE 6:**
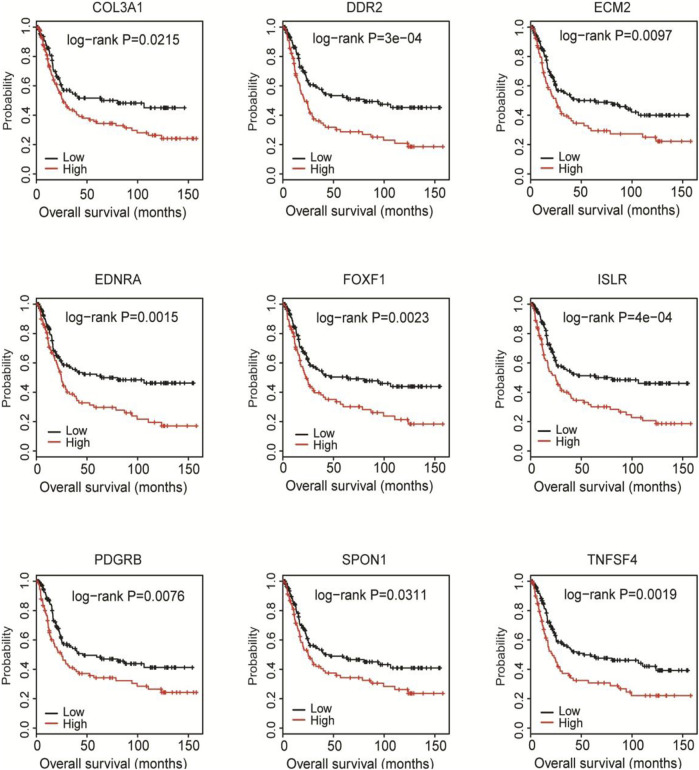
Validation of the prognostic value of the 9 hub genes in the GSE15460 cohort. The GSE15460 dataset was downloaded from the Gene Expression Omnibus (https://www.ncbi.nlm.nih.gov/geo/) as a validation cohort. Kaplan-Meier survival analysis was performed to examine the associations of 9 hub DEGs with the overall survival of the GSE15460 cohort.

Then, we validated the prognostic value of the 9 hub genes in the GSE15460 database. The Kaplan-Meier curves showed that the high expression of each gene was significantly correlated with shorter overall survival of the patients (all *p* < 0.05; Figure 6).

**FIGURE 7:**
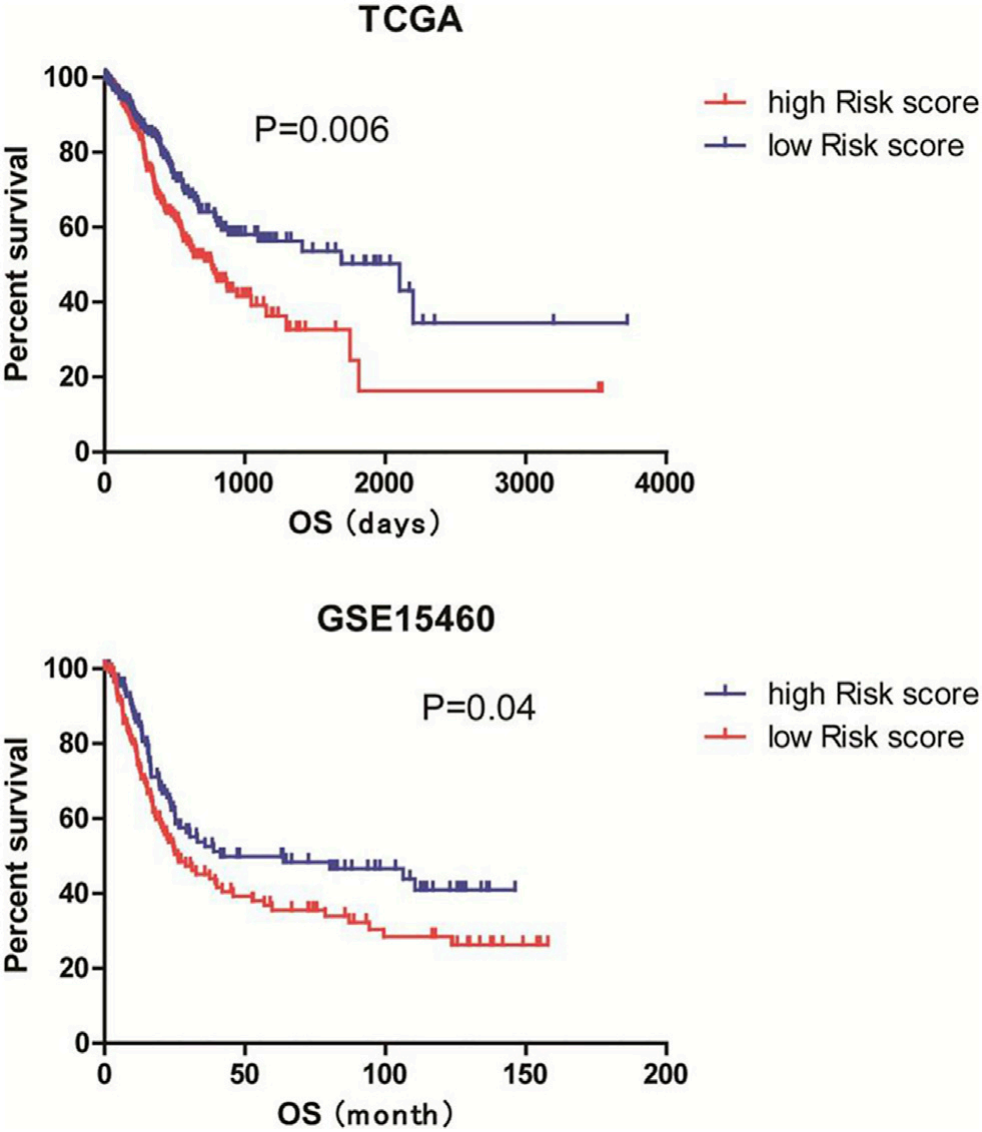
Establishment of a 9-gene-based risk model. The risk score of each patient in the TCGA and GSE15460 cohorts were calculated based on the Cox coefficient and expression level of each gene. Patients were divided into low and high risk score groups according to the mean risk scores. Kaplan-Meier survival analysis was performed to examine the associations of risk scores with the overall survival of the TCGA and GSE15460 cohorts.

**FIGURE 8:**
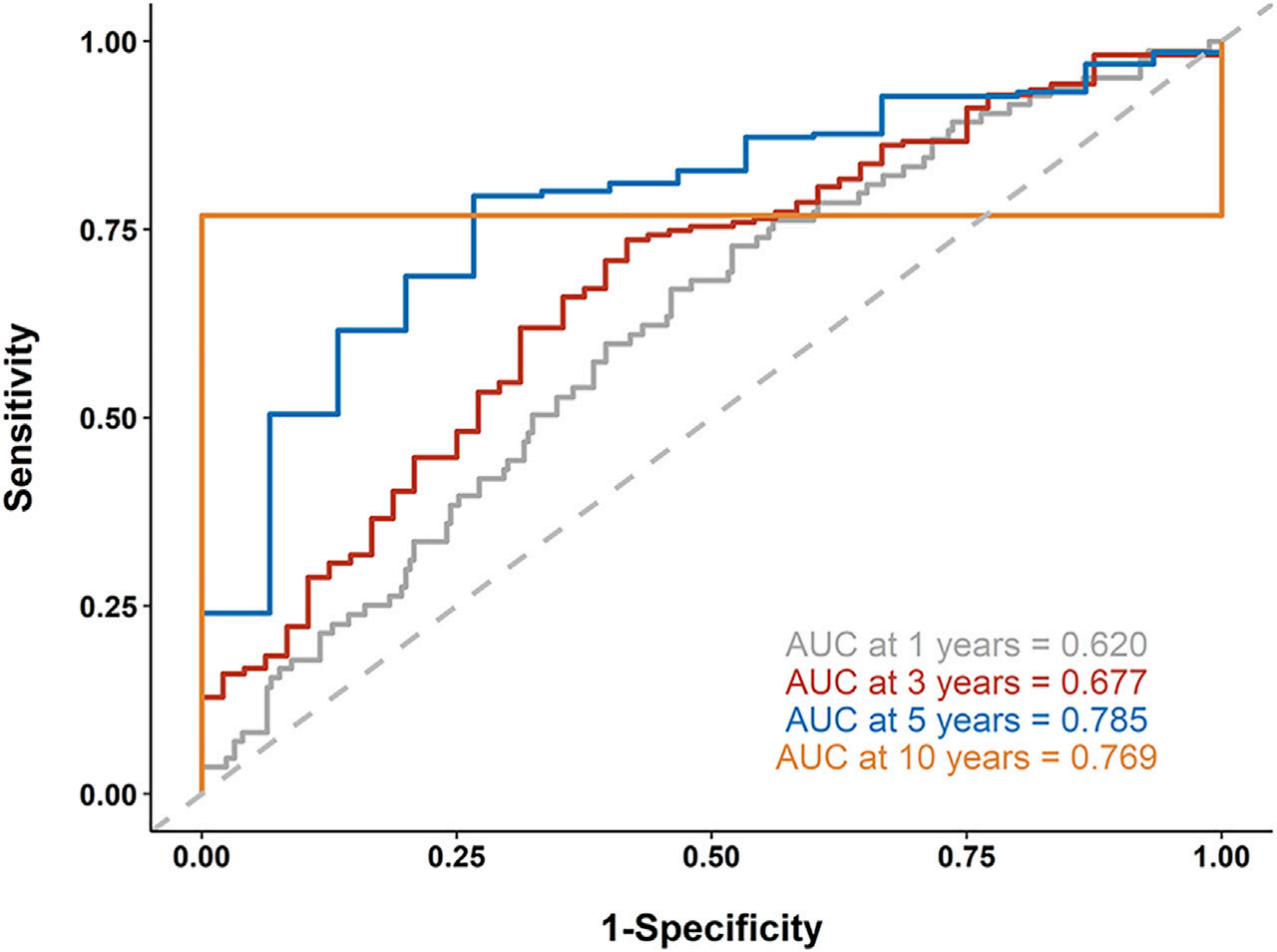
Receiver operating characteristic (ROC) analysis. A time-dependent ROC analysis was performed to evaluate the accuracy of the 9-gene-based risk model in 1-, 3-, 5-, and 10-years prediction horizons.

Next, we established a 9-gene-based prognostic risk signature and calculated the risk score of each patient in the TCGA and GSE15460 cohorts based on the Cox coefficient and expression level of each gene. Kaplan-Meier curves showed that patients with high risk scores had significantly shorter overall survival than those with low risk scores in both cohorts (both *p* < 0.05; Figure 7). Besides, a time-dependent ROC analysis yielded AUC values of 0.620, 0.677, 0.785, and 0.769 for 1-, 3-, 5-, and 10-years prediction horizons (Figure 8), respectively, indicating that the 9-gene-based prognostic model could accurately identify high-risk individuals within different time frames.

## Discussion

In this study, we hypothesized that some factors in the TME contribute to the poor response of GS subtype of gastric cancer to ICI therapy. To test our hypothesis, we compared the stromal and immune scores of different subtypes of gastric cancer in a TCGA cohort because infiltrating stromal and immune cells are major nontumor constituents of TME (Hanahan and Coussens, 2012; Arneth, 2019). We found that with comparable immune scores to the EBV subtype, the GS subtype had significantly higher immune scores than MSI and CIN subtypes, consistent with the findings showing that the GS subtype has enriched CD4^+^ T cells, macrophages, B cells, and TLS compared with other subtypes (Derks et al., 2020). We also noticed that the GS subtype had remarkably greater stromal scores than other subtypes, suggesting that the excessive infiltration of stromal components in GS tumors may reduce tumor response to ICI by suppressing the trafficking of TLS and immune cells into tumors (Şenbabaoğlu et al., 2016; Chen and Mellman, 2017). It has been reported that high stromal scores, but not immune scores, correlate with poor overall survivals of patients, serving as an independent prognostic factor in primary gastric cancer (Mao et al., 2020). Similar results were observed in our study. Thus, high stromal score might explain the low efficacy of ICI in the GS subtype and predict the poor prognosis of patients with gastric cancer.

ESTIMATE uses gene expression signatures to infer the fraction of stromal and immune components in TCGA tumor samples (Yoshihara et al., 2013). To apply it in clinical practice, we sought to construct a prognostic signature for gastric cancer based on ESTIMATE scores. Similar approaches have been applied in studies on different types of cancer (Ding Q. et al., 2020; Meng et al., 2020; Ma et al., 2021). After constructing the PPI network using the genes in the intersection between high stromal and immune score related DEGs, we identified 9 hub genes in the CXCL12 module, including COL3A1, DDR2, ECM2, EDNRA, FOXF1, ISLR, PDGRB, SPON1, and TNFSF4. The mRNA level of each gene was significantly, negatively correlated with the overall survival of the TCGA patients. The prognostic values of the 9 genes were further validated in the GSE15460 database. Based on these findings, we established a 9-gene-based prognostic risk signature and found that patients with high risk scores had significantly shorter overall survival than those with low risk scores. ROC analysis further suggests that the 9-gene-based prognostic model could accurately identify high-risk individuals within 1-, 3-, 5-, and 10-years prediction horizons.

Consistent with our results, studies have linked some components of the 9-gene risk signature with gastric cancer. For example, COL3A1 encoding Type III collagen is one of the upregulated hub DEGs in gastric cancer and predicts poor prognosis in patients ​based on the data downloaded from the GEO database (Shen et al., 2020). The polymorphism of transcription factor FOXF1 is associated with the prognosis of patients with gastric cancer (Matsusaka et al., 2018). ISLR, a member of the immunoglobulin superfamily, is highly expressed in gastric cancer tissue and associated with poor prognosis of patients (Li et al., 2020). PDGFRB that encodes platelet-derived growth factor receptor-*β* is overexpressed in gastric cancer tissue and associated with poor 5-years overall survival in patients with stage II/III gastric cancer receiving adjuvant chemotherapy with S-1, serving as an independent predictor of survival (Higuchi et al., 2017). SPON1 that encodes a secreted extracellular matrix glycoprotein is one of the DEGs in metastatic or poorly differentiated gastric cancer, being associated with tumor aggressiveness and poor prognosis of patients (Ding Y. et al., 2020). Taken together, these findings support the reliability of our 9-gene-based prognostic risk signature in predicting the prognosis of patient with gastric cancer. Besides, other genes that have not been linked with gastric cancer are promising candidates for gastric cancer research. However, further research such as certification in human tissues and cellular functional experiments are still warranted.

In conclusion, based on the ESTIMATE scores, we constructed a 9-gene-based prognostic risk signature to predict the prognosis for patients with gastric cancer. This finding may provide important information for prognostic stratification and therapeutic strategies for gastric cancer patients receiving ICI therapy.

## Data Availability

Publicly available datasets were analyzed in this study. This data can be found here: https://tcga-data.nci.nih.gov/tcga/, http://bioinformatics.mdanderson.org/estimate/ and https://www.ncbi.nlm.nih.gov/geo/.
